# A137 A CANADIAN PILOT STUDY: UNSEDATED TRANS-NASAL ENDOSCOPY AND BIOPSIES IN ADOLESCENTS WITH EOSINOPHILIC ESOPHAGITIS WITH VIRTUAL REALITY DISTRACTION

**DOI:** 10.1093/jcag/gwae059.137

**Published:** 2025-02-10

**Authors:** V Avinashi, B Greeley, E Greaney, J Bush, J Jacob

**Affiliations:** BC Children’s Hospital, Vancouver, BC, Canada; Digital Lab, BC Children’s Hospital, Vancouver, BC, Canada; Digital Lab, BC Children’s Hospital, Vancouver, BC, Canada; BC Children’s Hospital, Vancouver, BC, Canada; Digital Lab, BC Children’s Hospital, Vancouver, BC, Canada

## Abstract

**Background:**

Esoinophilic Esophagitis (EoE) is a chronic condition that requires endoscopy and biopsies for diagnosis and treatment evaluation. In pediatrics, anaesthesiology OR based sedation is standard practice, but it is resource intense, requires full fasting, and involves some risk. Trans-Nasal Endoscopy (TNE) with virtual reality (VR) distraction has successfully been used and evaluated at multiple Children’s Hospitals in the US. It uses thin endoscopes to obtain esophageal biopsies. This service is not available in Canada.

**Aims:**

Demonstrate that unsedated TNE:

- can be done safely in adolescents with EoE

- can be done reasonably comfortably

- tissue samples are adequate for histopathologic evaluation

**Methods:**

A pilot was launched spring 2024 at BC Children’s Hospital offering TNE with VR for EoE patients.

All TNE procedures were evaluated for any complications. Comfort / tolerability, was described using patient surveys including self-reported pain (Faces Pain Scale), worst pain during the procedure; anxiety (Visual Analogue Scale), VR and overall satisfaction from patients, caregivers, and staff. Heart rate was measured throughout. Biopsy samples were reviewed by a pathologist regarding size and quality.

**Results:**

7 teenage males have undergone and completed TNE, without complication. Mean procedure time was 9.6 minutes.

TNE resulted in some pain, which returned to baseline levels (Table 1). Patients’ (range = 5-6), caregivers’ (range = 6-7), and staffs’ (range = 6-7) scores demonstrated satisfaction with the overall procedure. All involved were very likely to recommend VR for the same procedure (range 9-10). All patients reported that they would do TNE with VR again.

Mean HR was elevated from baseline (68 BPM) compared to when the scope was inserted (78 BPM) and stayed elevated (74 BPM).

Mean # of biopsies obtained was 5.7. Average biopsy size in TNE was 0.16 cm (range: 0.1-0.4) compared to the patients’ historical EoE procedures of 0.22 cm (range: 0.1-0.6). One pathologist noted that orientation in one TNE sample was poor, due to small size. Pathologists felt the need to ink the tissues to ensure embedding. No samples were lost. All pathologists found TNE samples were sufficient for evaluation with eosinophil counts reported.

**Conclusions:**

Unsedated TNE with VR distraction at this early junction seems to be safe, reasonably well tolerated, and able to provide adequate specimens. Further research is needed to understand its feasibility in a more diverse sample.

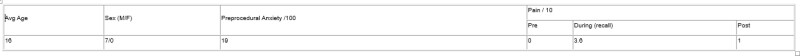

**Funding Agencies:**

Health Innovation Pathway Program: Minstry of Health BC; Equipment by Amacon(R)

